# National trends in venous thromboembolism incidence in Ireland (2015-2022): a retrospective analysis

**DOI:** 10.1016/j.rpth.2026.106798

**Published:** 2026-06-12

**Authors:** Richard Daly, Ciara Kirke, Cathal Hanley, Orla Healy, Barry Kevane, Fionnuala Ní Áinle, Maeve P. Crowley

**Affiliations:** 1 School of Medicine, College of Medicine and Health, University College Cork, Cork, Ireland; 2 Beaumont Hospital, Dublin, Ireland; 3 National Medication Safety Programme, Health Service Executive, Ireland; 4 Mater Misericordiae University Hospital, Dublin, Ireland; 5 National Quality & Patient Safety, Health Service Executive, Ireland; 6 School of Medicine, University College Dublin, Ireland; 7 Comprehensive Coagulation Centre, Cork University Hospital, Cork, Ireland

**Keywords:** health services administration, ICD-10, incidence, quality of health care, venous thromboembolism

## Abstract

**Background:**

Venous thromboembolism (VTE) affects millions globally, resulting in significant costs to patients and healthcare systems. Robust incidence data on VTE are limited, with a scarcity of national-level data in Ireland. Objectives: Determination of the crude VTE incidence in Ireland between 2015-2022; and assessment of VTE events during the COVID-19 during the pandemic, resulting in or occurring during a hospital admission.

**Methods:**

This was a retrospective observational study using Hospital In-patient Enquiry data obtained from hospital admissions in Ireland from 2015 to 2022. VTE events were identified using International Classification of Disease codes and categorized into primary (reason for admission), secondary (arising during admission), and hospital-acquired VTE. Census data were used to calculate crude incidence rates. Secondary rates were calculated per 1000 in-patient discharges and per 1000 elective hip/knee arthroplasties.

**Results:**

Between 2015 and 2022, 51,123 adult VTE cases were recorded, with a mean annual crude incidence of 172 per 100,000 population. The mean adult primary VTE crude incidence was 98 per 100,000 and mean annual hospital–acquired VTE rate was 9.9 per 1000 discharges. Of the primary VTE cases, 15,284 (52%) were in females. A total of 218 pediatric VTE cases were identified (0.23 per 10,000 population). Pregnancy-associated VTE accounted for 2.4 VTE events per 1000 live births. The mean incidence of VTE was 11 per 1000 elective hip/knee arthroplasties.

**Conclusion:**

This national report of VTE incidence in Ireland provides data on primary and secondary VTE trends. From 2020 to 2022, increases in pediatric VTE, adult pulmonary embolism, and pregnancy-associated VTE and decreases in adult deep vein thrombosis were observed, in part due to the COVID-19 pandemic. This forms essential baseline data to inform VTE prevention, surveillance, further research, and health policy.

## Essentials

• VTE, a major cause of morbidity and mortality, is lacking robust national-level incidence data in Ireland.
• Between 2015 and 2022, Ireland’s mean annual crude VTE incidence was 172 per 100,000 population.
• VTE rates rose during the COVID-19 pandemic.
• This research will inform VTE prevention, surveillance, further research, and health policy.

## Graphical abstract

**Figure:**
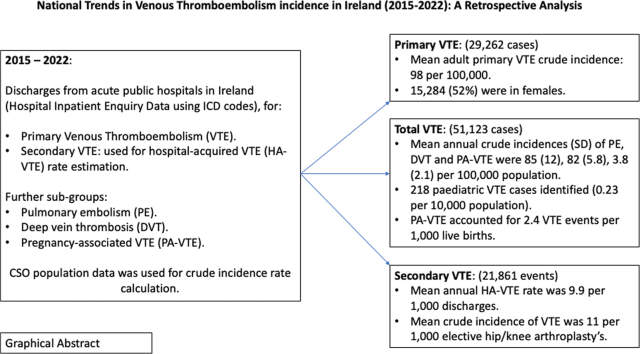

## 1 Introduction

Venous thromboembolism (VTE), comprising deep vein thrombosis (DVT) and pulmonary embolism (PE), annually impacts millions of people [1]. While the disease burden is globally well understood, there is a paucity of Irish VTE data compared with other jurisdictions [2]. Availability of comprehensive information about VTE incidence and mortality within a healthcare system is crucial for effective planning and resource allocation, particularly for underrecognized and vulnerable groups [2].

Well-recognized risk factors for VTE include, but are not limited to, history of VTE or thrombophilia, prolonged immobilization, pregnancy, trauma, surgery, cancer, hormonal treatment, older age, congestive heart disease, respiratory failure, obesity, and COVID-19 [[3], [4], [5]]. Thrombosis is the leading cause of direct maternal death in the United Kingdom and Ireland [6,7]. The long-term sequelae of VTE are wide-ranging, including post–thrombotic syndrome, post-PE syndrome, and chronic thromboembolic pulmonary hypertension. Long-term complications can result in significant negative impacts on patients’ quality of life, increased healthcare utilization, and economic burdens for both the patient and society as a whole [8].

Hospital-acquired VTE (HA-VTE) is defined as a VTE that occurs either during or up to 90 days after hospitalization [1]. Hospitalized patients often present with a number of the risk factors described above, exacerbated by hospital-specific factors such as immobility, invasive procedures, inflammation, and infection. In an Irish VTE report, HA-VTE accounted for at least 47% of VTE cases, thereby posing a substantial and potentially preventable patient safety issue [2]. Thrombotic events in hospitalized children are increasingly being recognized, but our understanding of the effects of VTE in children is limited [9]. Despite widespread implementation of VTE risk assessment and thromboprophylaxis strategies, HA-VTE continues to be an issue.

Accurate VTE incidence data can be challenging to obtain. Data can be acquired from a variety of sources such as electronic health records, hospital discharge databases, insurance claims surveys, and cohort studies, depending on how data are collected and managed within the healthcare system in question. Many of these systems were not designed to collect incidence data but as they are embedded into healthcare systems, they can be useful tools for disease incidence estimation.

Estimating VTE incidence rates at a population level typically involves using administrative codes for VTE. The International Classification of Diseases (ICD) codes are the most commonly used method for this purpose [10]. Significant variability exists in the individual codes used in publications [2,11,12]. An early aim of the National Clinical Program in VTE was to make data on VTE incidence in Ireland available to guide resource allocation and influence health policy.

The primary aim of this study was to determine the crude incidence of VTE in Ireland between 2015 and 2022. Secondary aims were to estimate the incidence and proportion of PE, DVT, and pregnancy-associated VTE (PA-VTE) resulting in or occurring during a hospital admission and to investigate associations between VTE incidence and the COVID-19 pandemic.

## 2 Methods

A retrospective observational study design was used.

### 2.1 Ethical approval

Ethical approval was not required because our analysis relied on pre-existing datasets containing anonymized information managed by the Health Service Executive (HSE). Individual patient identities and location were not accessible, ensuring anonymity.

### 2.2 Study population

The study population comprised the people served by acute public hospitals in the Republic of Ireland (hereafter referred to as Ireland). Retrospective data pertaining to patients admitted to hospital over an 8-year predetermined period from January 2015 to December 2022 were included.

### 2.3 Data sources

The estimated population of this study (based on census data) increased from 4,761,865 in 2016 to 5,419,139 in 2022 (source: Central Statistics Office [CSO]). Population projections were used in intervening years [13].

Consolidated data were obtained from the Hospital In-patient Enquiry (HIPE) Healthcare Pricing Office (HPO). HIPE is a health information system that collects data on discharges from acute public hospitals in Ireland, including administrative, clinical and demographic data [14].

Each hospital discharge is recorded in HIPE as being associated with 1 primary diagnosis and ≥1 secondary diagnosis coded according to ICD [14]. The ICD is updated every 4 to 5 years; in Ireland, the International Statistical Classification of Diseases and Related Health Problems, Tenth Revision, Australian Modification (ICD-10-AM) for diagnosis codes has been used for HIPE discharges since January 1, 2020; prior to that, the Eighth Revision of the ICD was used (2015-2019).

HIPE data underwent input and verification by trained HIPE coders at the hospital level upon patient discharge, utilizing standardized discharge forms completed by the responsible clinician. After input and verification, the data were subject to further validation by the centralized HSE HIPE HPO. We liaised closely with HIPE managers to ensure the quality of our analysis [15].

The data analyzed in this study were contemporaneously collected by HIPE from 2015 to 2022 and received by the investigators in 2024 and 2025.

### 2.4 VTE case identification

Cases of VTE were identified on the basis of ICD-8 (2015-2019) and ICD-10-AM (2020-2022) discharge diagnostic codes [16]. Codes were grouped into those pertaining to PE, DVT, and PA-VTE events (Table 1). There are some differences in the code descriptions between editions; however, they did not impact our data. Code I80.2 (Phlebitis and thrombophlebitis of other deep vessels of lower extremities) is present in both ICD-8 and ICD-10-AM and encompasses all relevant subcodes, including I80.22 (Phlebitis and thrombophlebitis of popliteal vein), which is specific to ICD-10-AM.

**Table 1:** ICD-8 and ICD-10-AM discharge diagnostic codes for VTE.

PE	I26.0	PE with mention of acute cor pulmonale
	I26.9	PE without mention of acute cor pulmonale
	I80.1	Phlebitis and thrombophlebitis of femoral vein
	I80.2	Phlebitis and thrombophlebitis of other deep vessels of lower extremities
	I80.3	Phlebitis and thrombophlebitis of lower extremities, unspecified
	I80.8	Phlebitis and thrombophlebitis of other sites
DVT	I80.9	Phlebitis and thrombophlebitis of unspecified site
	I82.2	Embolism and thrombosis of vena cava
	I82.8	Embolism and thrombosis of other specified veins
	I82.9	Embolism and thrombosis of unspecified vein
	O08.2	Embolism following abortion and ectopic and molar pregnancy
	O22.3	Deep phlebothrombosis in pregnancy
	O22.9	Venous complication in pregnancy, unspecified
PA-VTE	O87.1	Deep phlebothrombosis in the puerperium
	O87.9	Venous complication in the puerperium, unspecified
	O88.2	Obstetric blood clot embolism

DVT, deep vein thrombosis; ICD-8, International Classification of Diseases, Eighth Revision; ICD-10-AM, International Statistical Classification of Diseases and Related Health Problems, Tenth Revision, Australian Modification PA, pregnancy-associated; PE, pulmonary embolism; VTE, venous thromboembolism.

“Primary VTE” was defined as a VTE coded in HIPE as being the primary reason for the patient’s admission to hospital. VTE managed through ambulatory care pathways were not included in the analysis, as they are not available via HIPE.

“Secondary VTE” was defined by a VTE event coded in HIPE as occurring during the hospital admission but not being the primary reason for the patient’s admission. Discharges with >1 overnight (ie, ≥ 2 days) stay were included. Data on “secondary” VTE can be viewed as an indirect estimate of HA-VTE. This is an approximation and likely overestimation of HA-VTE [16].

Further examination of the population (using CSO data) included groups aged ≥18 years, <18 years, sex (male or female, according to their reproductive organs and functions based on chromosomal complement), all live births, and those undergoing a hip/knee replacement [13]. Annual crude incidence was determined using these data [17]. A calculation of annual national crude incidence rates of VTE involved dividing the number of hospital discharges with a primary VTE diagnosis by the Irish population (as reported by the CSO), for each corresponding year, to determine the estimated incidence rate. Rates are expressed per 100,000 population. Cumulative incidence was not the best manner to present the data, as our dataset does not track individuals over time.

The crude incidence of primary DVT, PE, and PA-VTE (where the VTE was recorded as the reason for admission) was calculated per 100,000 population for adults aged ≥ 18 years and per 10,000 population for those <18 years, denoted pediatric.

PA-VTE was defined by select pregnancy-related venous conditions as per ICD (Table 1). The incidence of PA-VTE was calculated on a population level and using the denominator of all births as reported by the CSO for each year. This denotes all live births. Note, in the case of a twins, there would be 2 birth registrations derived from that delivery event so both live births are counted [13]. Pediatric VTE cases were defined as those occurring in those aged <18 years.

The HA-VTE rate (mean annual occurrence of VTE associated with hospitalization) from 2015 to 2022 was estimated by calculating the number of secondary VTE events recorded per 1000 in-patient discharges with ≥1 overnight stay. The numerator was the number of adult in-patient discharges with a “secondary diagnosis of VTE,” defined as any of the ICD codes listed in Table 1. The denominator was the total number of adult discharges in the reference year with ≥1 overnight stay in an in-patient bed. The mean of the annual occurrence rates was calculated to determine the HA-VTE rate for 2015-2022. For elective hip/knee replacement surgeries, similar to the Organisation for Economic Co-operation and Development (OECD) indicator, patients aged >15 years were included [18].

### 2.4 Statistical analysis

Descriptive statistics elucidating primary and secondary VTE trends were generated, including mean, SD, 95% CIs, median, and IQR where relevant. Data were processed by HIPE using SAS software. The association between variables was conducted upon importing the data into Excel and GraphPad Prism 10.

## 3 Results

Between 2015 and 2022, a total of 51,123 cases of VTE were recorded in adults resulting in or occurring during hospital admissions in Ireland, with a mean annual crude incidence of 172 (SD 11) per 100,000 population (Table 2). These consisted of 29,262 primary VTE and 21,861 secondary VTE cases. The mean annual crude incidences (SD) of PE, DVT, and PA-VTE were 85 (12), 82 (5.8), 3.8 (2.1) per 100,000 population (Figure).

**Table 2:** Number and crude incidence of primary, secondary, and total VTE for 2015-2022.

Year	Population	PE		DVT		PA-VTE		Total VTE	
		No.	Incidence	No.	Incidence	No.	Incidence	No.	Incidence
Primary									
2015	3,504,048	1529	43.6	1815	51.8	55	1.6	3399	97
2016	3,550,340	1452	40.9	1837	51.7	75	2.1	3364	94.8
2017	3,613,126	1616	44.7	1794	49.7	69	1.9	3479	98
2018	3,677,243	1618	44	1963	53.4	50	1.4	3631	96.3
2019	3,746,580	1597	42.6	1887	50.4	55	1.5	3539	94.5
2020	3,816,989	1841	48.2	1751	45.9	86	2.3	3678	96.4
2021	3,866,543	2147	55.5	1847	47.8	154	3.9	4148	107.3
2022	3,962,442	1879	47.4	2031	51.3	114	2.1	4024	101.6
Total		13,679		14,925		658		29,262	
Mean (SD)	3,717,164 (159,898)	1710 (229)	46 (4.6)	1866 (92)	50 (2.4)	82 (35.7)	2.1 (0.8)	3658 (258)	98 (4.3)
95% CI	3,583,486-3,850,842	1519-1901	42-49.7	1789-1943	48.2-58.3	52.4-112	1.4-2.8	3418-3897	94.7-102
Secondary									
2015	3,504,048	1168	33.3	1173	33.5	27	0.8	2368	67.6
2016	3,550,340	1198	33.7	1190	33.5	28	0.8	2416	68.1
2017	3,613,126	1254	34.7	1212	33.5	30	0.8	2496	75.6
2018	3,677,243	1383	37.6	1275	34.7	18	0.5	2676	72.3
2019	3,746,580	1239	33.1	1430	38.2	31	0.8	2700	72.1
2020	3,816,989	1573	41.2	997	26.1	112	2.9	2682	70.3
2021	3,866,543	2083	53.9	1107	28.6	149	3.9	3339	86.4
2022	3,962,442	1888	47.6	1187	29.9	109	2.0	3184	83.7
Total		11,786		9571		504		21,861	
Mean (SD)	3,717,164 (159,898)	1473 (345)	39 (7.7)	1196 (125)	32 (3.8)	63 (51.5)	1.6 (1.2)	2733 (352)	75 (7)
95% CI	3,583,486-3,850,842	1185-1762	32.9-45.8	1092-1301	29-35.5	19.9-106	0.5-2.6	2438-3027	68.6-80.4
Total									
2015	3,504,048	2697	76.9	2988	85.3	82	2.3	5767	164.6
2016	3,550,340	2650	74.6	3027	85.2	103	2.9	5780	162.9
2017	3,613,126	2870	79.4	3006	83.2	99	2.7	5975	165.4
2018	3,677,243	3001	81.6	3238	88.1	68	1.8	6307	171.5
2019	3,746,580	2836	75.7	3317	88.5	86	2.3	6239	166.5
2020	3,816,989	3414	89.4	2748	72	198	5.2	6360	166.6
2021	3,866,543	4230	109.4	2954	76.4	303	7.8	7487	193.6
2022	3,962,442	3767	95.1	3218	81.2	223	5.6	7208	181.9
Total		25,465		24,496		1,162		51,123	
Mean (SD)	3,717,164 (159,898)	3183 (568.5)	85 (12)	3062 (185.3)	82 (5.8)	145 (85)	3.8 (2.1)	6390 (636.5)	172 (11)
95% CI	3,583,486-3,850,842	2708-3658	75-95	2907-3217	78 -87	74-217	2-5.6	5858-6922	163-181

Annual and combined (2015-2022) total with mean (SD) and 95% CI per 100,000 population aged ≥ 18 years are shown. For PA-VTE, admissions to maternity hospitals were excluded; however, cases assigned a secondary PA-VTE code by HIPE coders in acute hospitals were included.

DVT, deep vein thrombosis; HIPE, Hospital In-patient Enquiry; PA, pregnancy-associated; PE, pulmonary embolism; VTE, venous thromboembolism.

**Figure:**
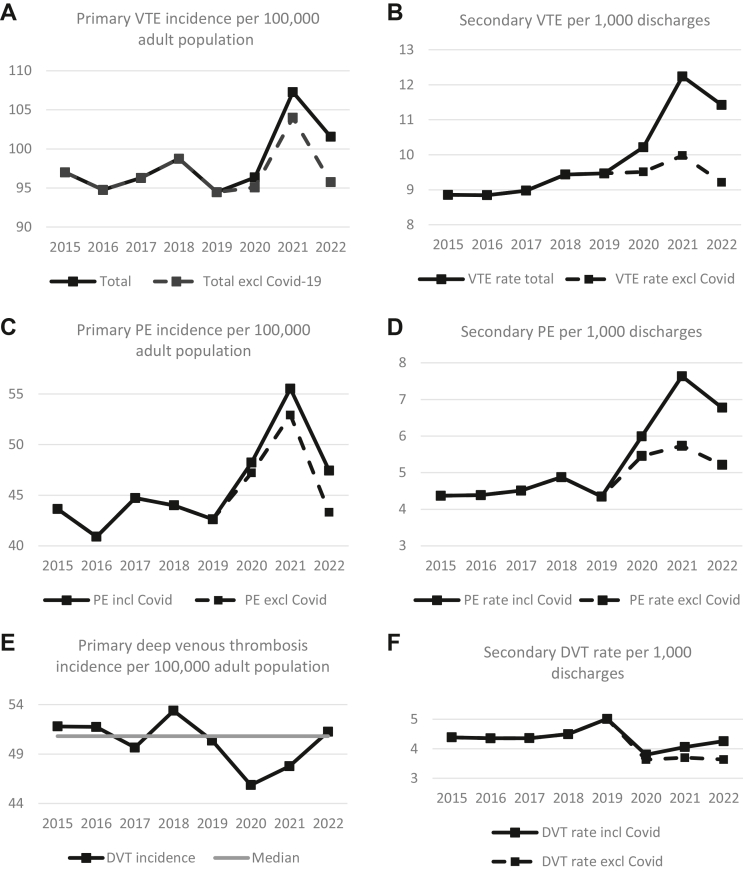

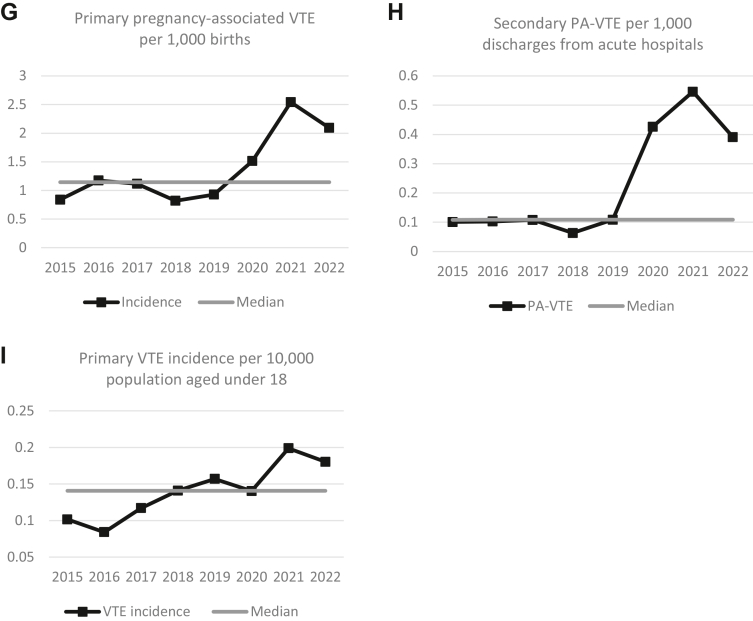
Trends for VTE occurring between 2015 and 2022. Primary VTE in adults (A), secondary VTE (B), primary PE in adults (C), secondary PE (D), primary DVT in adults (E), secondary DVT (F), primary PA-VTE (G), secondary PA-VTE (H), and primary pediatric VTE (I). For (G) and (H), the reason the data is not stratified by COVID-19 status is due to the low numbers of PA-VTE resulting in anonymization; however, for primary DVT (E), the data was suppressed to prevent to disclosure of other suppressed cells. Incidence denotes crude incidence. DVT, deep vein thrombosis; PA, pregnancy-associated; PE, pulmonary embolism; VTE, venous thromboembolism

A total of 218 cases of VTE were recorded in the population aged <18 years. Of these, 135 cases of primary VTE were recorded. Secondary pediatric VTE data was not available, except for neonates, and 83 cases of secondary VTE (the latter in neonates only) were recorded. The mean annual crude incidence of VTE was 0.23 per 10,000 population aged <18 years.

### 3.1 Primary VTE

A total of 29,262 cases of primary VTE were recorded in adults aged ≥ 18 years, an annual mean of 3658 (SD 287; 95% CI: 3418-3897) (Table 2). The mean adult annual crude incidence of primary VTE admissions from 2015 to 2022 was 98 (SD 4.3) per 100,000 population (Table 2). Primary VTE affected more females (52.2%) than males, giving an incidence of 100.7 and 96 per 100,000, respectively.

A total of 13,679 primary PE diagnoses were recorded in adults, annual mean 1710 (SD 229; 95% CI: 1551-1868) (Figure; Table 2). Among these, 15.8% (270 cases) were associated with acute cor pulmonale. The mean (SD) annual crude incidence of primary PE was 45.9 (4.6) per 100,000 population from 2015 to 2022; 9.8% (1344) cases were admitted to the intensive care unit (ICU), with a median length of stay (LOS) of 13.4 days in the hospital (IQR: 12.2-14.3) and 4.5 days in the ICU (IQR: 4.3-4.7) (Supplementary Table). There were 413 deaths of patients in whom primary PE was listed as the primary diagnosis, a mean of 52 (SD 7.4) deaths per annum.

In adults, 14,925 DVT cases were reported, with an annual mean of 1866 cases (SD 92; 95% CI: 1789-1983). The median LOS was 3.8 days (IQR: 3.7-4.0). The mean annual primary DVT crude incidence was 50.2 (SD 2.4) per 100,000 population from 2015 to 2022. In total, 84 deaths were recorded (0.6%), with an annual mean of 11 deaths (SD 3) (Supplementary Table).

PA-VTE accounted for 2% (658) of primary VTE events, with a mean of 82 cases per year (SD 36; 95% CI: 52-112) (Table 2). The median LOS was 3.1 days (IQR: 2.4-3.8) (Supplementary Table). The mean annual crude incidence was 2.1 cases (SD 0.8) per 100,000 population (Table 2). A rate of 1.4 cases of primary PA-VTE (SD 0.6) was recorded per 1000 births from 2015 to 2022 (Table 3 [18]). The mean age of patients was 32.7 years (SD 1.0; 95% CI: 31.8-33.5). No deaths, ICU stays. or hemorrhages were recorded.

**Table 3:** Number and rate of primary, secondary and total VTE per 1000 live births.

Year	Live births [18]	Primary VTE	Rate per 1000 live births	Secondary VTE^a^	Rate per 1000 live births	Total VTE^a^	Rate per 1000 live births
2015	65,536	55	0.8	27	0.5	82	1.5
2016	63,841	75	1.2	28	0.5	106	1.7
2017	61,824	69	1.1	30	0.5	99	1.7
2018	61,022	50	0.8	18	0.3	68	1.1
2019	59,294	55	0.9	31	0.5	86	1.4
2020	56,812	86	1.5	112	1.8	198	3.2
2021	60,575	154	2.5	149	2.3	303	4.7
2022	54,483	114	2.1	109	1.7	223	3.4
Total	483,387	658	1.4	504	1.0	1162	2.4
Mean (SD)	60,423 (3581)	82 (36)	1.4 (0.6)	63 (51.5)	1.0 (0.8)	145 (86)	2.4 (1.3)
95% CI	57,430-63,417	52.4-112.1	0.8-1.9	19.9-106.1	0.4-1.7	74.4-216.9	1.3-3.4

Annual and combined (2015-2022) total with mean (SD) and 95% CI are shown.

HIPE, Hospital In-patient Enquiry; PA, pregnancy-associated; VTE, venous thromboembolism.

a For PA-VTE, admissions to maternity hospitals were excluded; however, cases assigned a secondary PA-VTE code by HIPE coders in acute hospitals were included.

For pediatric VTE, from 2015 to 2022, a total of 135 pediatric VTE cases resulting in admission to hospital were recorded, with a mean of 17 cases per year (SD 5; 95% CI: 13-21) (Figure). The mean annual primary pediatric VTE crude incidence was 0.14 cases per 10,000 population (SD 0.04) during this period. There were no recorded PA-VTE events in the pediatric group.

In total, 497 deaths were recorded (annual mean 62; SD 9.4) in patients for whom VTE was the primary cause of admission. Primary VTE therefore was recorded in association with (but not necessarily causative of) 0.2% of deaths in Ireland per annum overall during this time period [13].

Of the cases of primary VTE, 406 had an associated secondary diagnosis of COVID-19 (2020-2022 annual mean: 135, SD 91; min-max: 49-230). Of the COVID-19 related cases, 303 (75%) had PE as primary diagnoses (annual mean 101). In the PA-VTE group, there were no COVID-19 cases reported in 2020, and in 2021-2022, the data was suppressed, as there were <5 cases each year.

### 3.2 Secondary VTE

From 2015 to 2022, 2,201,456 discharges were recorded in adults, an annual mean of 275,182 (SD 7752). A total of 21,861 secondary VTE events were recorded, with a mean of 2733 cases per year (SD 352; 95% CI: 2510-2955). The mean annual crude incidence of secondary VTE in adults was 9.9 (SD 1.3) per 100,000 population (Table 4).

**Table 4:** Number and rate of secondary VTE per 1000 discharges from acute hospitals in adults aged ≥ 18 years.

Year	PE	DVT	PA-VTE	Total	Discharges	PE rate	DVT rate	PA-VTE rate	Total rate
2015	1168	1173	27	2368	267,411	4.4	4.4	0.1	8.9
2016	1198	1190	28	2416	273,177	4.4	4.4	0.1	8.8
2017	1254	1212	30	2496	278,082	4.5	4.4	0.1	9.0
2018	1383	1275	18	2676	283,579	4.9	4.5	0.1	9.4
2019	1239	1430	31	2700	285,173	4.3	5.0	0.1	9.5
2020	1573	997	112	2682	262,544	6.0	3.8	0.4	10.2
2021	2083	1107	149	3339	272,821	7.6	4.1	0.5	12.2
2022	1888	1187	109	3184	278,669	6.8	4.3	0.4	11.4
Total	11,786	9,571	504	21,861	2,201,456	42.9	34.7	1.8	79.5
Mean	1473.3	1196.4	63.0	2732.6	275,182	5.4	4.3	0.2	9.9
SD	345.1	125.0	51.5	352.0	7751.5	1.3	0.4	0.2	1.3
95% CI	1185-1762	1092-1301	19.9-106.1	2438-3027	268,702-281,662	4.3-6.4	4.1-4.6	0.1-0.4	8.9-10.9

DVT, deep vein thrombosis; PA, pregnancy-associated; PE, pulmonary embolism; VTE, venous thromboembolism.

Of these, 53.9% (11,786 cases) were secondary PE in adults, with an annual mean of 1473 cases (SD 345; 95% CI: 1250-1696). Secondary DVT accounted for 43.8% (9571 cases) with an annual mean of 1196 cases (SD 125; 95% CI: 1110-1283). Secondary PA-VTE comprised 2.3% (504 cases) with an annual mean of 63 cases (SD 52; 95% CI: 36-99) (Table 4).

For secondary VTE, 83 cases of DVT occurred in neonates, with an annual mean of 10.4 cases (SD 3.7; 95% CI: 7.3-13.4). Data for older children was unavailable for secondary VTE.

The national mean annual HA-VTE rate per 1000 adult discharges was 9.9 (SD 1.3).

From 2020 to 2022, 1371 cases of secondary VTE had a concomitant COVID-19 secondary diagnosis, a mean of 457 cases per annum (SD 243; min-max: 177-607). PE accounted for 1096 (77%) cases (annual mean: 365, SD 199). DVT accounted for 316 (22%) cases; (annual mean: 105, SD 65.3).

In our orthopedic cohort (elective hip/knee arthroplasty), 347 cases (annual mean: 43, SD 6; 95% CI: 39-48) of HA-VTE were recorded. Of the cases, 194 (56%) were PE (annual mean: 24, SD 5.0); 153 cases (44%) were DVT (annual mean: 19; SD 5.3; 95% CI: 15-23), and there were no PA-VTE events recorded in this group. The crude incidence of hip/knee arthroplasty-related VTE was 11 per 1,000 elective hip/knee arthroplasty patients for 2015-2022.

### 3.3 Trends over time

Figure illustrates the trends in the crude incidence of primary VTE and rate of secondary VTE over time. The period 2020-2022 showed marked differences from earlier years. The crude incidence of primary VTE, primary PE, and primary PA-VTE in adults and primary pediatric VTE increased, particularly in 2021, and the crude incidence of primary DVT was lower in 2020 and 2021. The rate of secondary VTE followed a similar pattern, with PE, PA-VTE, and overall VTE increasing from 2020 to 2022, and DVT rates were lower in 2020 and 2021. These changes were less pronounced but persisted when cases with a concomitant secondary diagnosis of COVID-19 were excluded (Figure A–F).

## 4 Discussion

This study represents a national report on the crude incidence of primary VTE in Ireland and provides additional exploratory data on in-patient primary and secondary VTE trends. Between 2015 and 2022, 51,123 cases of VTE were recorded in adults, a mean annual crude incidence of 172 (SD 11) per 100,000 population. This is similar to previously published international data for high income countries of 1 to 2 cases per 1000 population [1]. It is similar to the incidence reported in a recent Irish regional study (1.44 per 1000 population per annum) [2].

An additional 218 cases of pediatric VTE were recorded resulting in-hospital admission or occurring during admission in neonates, representing a mean annual crude incidence of 0.23 per 10,000 population in individuals aged <18 years.

Adult females were more likely to develop a primary VTE than males, consistent with data from recent epidemiologic studies that report a lifetime risk of 9.3% for women and 8.1% for men [19]. The annual crude incidences of VTE events appeared stable until 2019. In 2020, 2021, and 2022, rises in the rates of primary and secondary PE, PA-VTE, and primary pediatric VTE were noted and decreases in the rates of primary and secondary DVT. COVID-19 co-diagnosis accounted for some but not all of these changes. A UK study reported similar findings of increased VTE hospitalization in 2020-2022 [20]. Their time series from 1998 to 2022 identified an upward trend in PE hospitalizations and a decrease in DVT hospitalizations [20].

COVID-19-associated coagulopathy is a complex dysregulation of multiple pathways [21]. A total of 1777 cases of VTE were coded with an associated diagnosis of COVID-19. As the HIPE system does not differentiate between SARS-CoV-2 positivity and the clinical syndrome of COVID-19, it is likely that SARS-CoV-2 positivity may have been an incidental finding in a proportion of cases. The peak primary and secondary VTE crude incidence during this study period occurred in 2021 and 2022, coinciding with a high community and hospital burden of COVID-19 at several time points. Of note, in 2020, hospital activity affected the denominator, rendering interpretation of the 2020 VTE data quite challenging.

DVT accounted for only 21% of cases of VTE in adults with a COVID-19 co-diagnosis but 48% of overall VTE cases. The reduction in the rates of primary and secondary DVT in 2020 and 2021 are likely contributed to by greater management outside of the hospital setting due to concerns about COVID-19 transmission in-hospital. Although data were not available where DVT was treated in an ambulatory setting, we believe that this report is a valuable contribution to our national knowledge-base, which will help to plan services to reduce the burden of hospital admission for primary DVT.

PA-VTE is attributed to the hypercoagulable state of pregnancy [22]. The mean annual crude incidence of PA-VTE was determined to be 2.4 (SD 1.3) cases per 1000 live births per annum, despite our data excluding secondary VTE in maternity admissions and maternity hospitals. Pregnancy increases the risk of VTE by 4- to 5-fold, although the absolute risk is reported to be low (<1 per 1000 pregnancies) [6,23]. Our rates were higher on average, although the baseline rates up to 2019 were in line with international literature [6,[23], [24], [25]]. Multiple factors have been suggested as increasing the rates of PA-VTE (eg, increases in fertility treatments, premature births, and cesarean sections) [6,25,26]. The timing and extent of the dramatically increased incidence of PA-VTE in 2020-2022 in our study coincides with population levels of COVID-19 infection, although few women were recorded as having a co-diagnosis of COVID-19. A population study in Sweden and Norway found pregnant women to have an increased incidence rate of VTE if they had a COVID-19 infection compared with noninfected (adjusted overall hazard ratio 1.26), with a higher risk postpartum [27]. Increased risk was highest in the first 2 weeks postinfection but remained raised at 12 weeks.

In Ireland, all deaths of women while pregnant or within 42 days of the end of pregnancy are formally investigated. Although no deaths were recorded in HIPE as being related to PA-VTE, a review of the triennial Confidential Enquiries into Maternal Deaths Reports 2015-2017 and 2018-2020 revealed 2 maternal deaths related to VTE between 2018 and 2020 [6,[28], [29], [30]]. It is likely that these deaths were coded as PE-related deaths rather than PA-VTE-related deaths. The manner in which PA-VTE is coded by HIPE requires examination. These data will guide resource allocation to address pregnancy-specific challenges, which include diagnostic imaging choice, prioritization of clinical trials, and the necessity for timely multidisciplinary management [24].

We identified 218 cases of pediatric VTE, of which 135 and 83 were primary and secondary VTE events, respectively. VTE in pediatric patients are rare [31]. An increase in the crude incidence of primary VTE was noted in 2021 and 2022. Overall numbers remained small, and DVT was more commonly recorded than PE. In particular, all secondary VTE events occurring in neonates were DVTs. The absence of secondary VTE data for pediatric patients, with the exception of neonates, renders comparisons more challenging; however, the available VTE crude incidence in our primary and secondary pediatric cohort for this period was 0.23 per 10,000 population. This compares with 0.07 to 0.49 [32] and 0.14 [33] per 10,000 population in international studies. For 2015-2022, secondary VTE incidence in neonates was 1.7 per 10,000 population aged <1 year. Comparison is challenging due to a paucity of data and differing denominators, eg, a neonatal VTE rate of 75 per 10,000 hospital admissions of children <28 days old has been reported [34].

The term “secondary” VTE diagnosis was used to estimate the occurrence of HA-VTE, aligning with current data collection methods in Ireland. Published literature suggests that a substantial portion of HA-VTE events occur after hospital discharge and thus would typically be categorized as “primary” VTE events in our data collection systems if this results in a hospital admission [35]. Moreover, accurate determination of both primary and secondary VTE using ICD codes alone is known to be challenging but often represents the only pragmatic solution to data gathering at a national-level, where case validation would be unfeasible.

The mean annual secondary VTE rate of 9.9 (SD 1.3) per 1000 discharges are similar to a cohort study that identified that 1.2% of non-ICU medical patients developed HA-VTE during or after admission [36], although our study included ICU patients. Our rate is higher than the 4 HA-VTE cases per 1000 discharges reported in an Irish study [37]. However, a large tertiary referral center in Ireland reported a positive predictive value (PPV) with the same HA-VTE methodology used in our study of 0.37 [16]. This was similar to other international reports, with a range from 30% to 80% [12,16,38,39]. When the PPV is applied to our data, this gives a figure similar to other studies [16]. Our HA-VTE rate, using data from 2015 to 2022, is also lower than the rate reported in a recent Irish study, 11.38 per 1000 discharges in 2022 [16]. However, applying the same methodology for 2022 only, our results are comparable at 11.4 per 1000 discharges [16]. This is reflected in our data, as 2021 and 2022 rates were higher than in other years. These variations underscore the complexity and heterogeneity of HA-VTE rates reported across different studies and populations. Quality improvement measures to improve the PPV of these codes in Ireland are underway [2,16].

The rate of HA-VTE after elective hip/knee replacement surgeries was 11 per 1000 patients from 2015 to 2022. Similar to the OECD indicator, patients aged ≥ 15 years were included. This is higher than postoperative VTE rates in Ireland (597/100,000) and the OECD (529/100,000) after hip and knee replacements [18], possibly reflecting the lower PPV of our data. These data reflect secondary VTE occurring during admission and do not include VTE arising later, resulting in admission to the same or a different hospital [40]. The introduction of eHealth initiatives in Ireland is likely to improve the ability to track patient progress after discharge.

The original reported goals of the HIPE data initiative were to establish a standard minimum dataset concerning in-patient morbidity and mortality, to streamline the analysis of hospital activity, and to enable cross-country comparisons [14,41]. While it is clear that HIPE data do not accurately quantify HA-VTE, a complexity also reported by our closest geographical neighbors in the UK [42,43], this remains the most economically viable way to estimate VTE incidence at a population level at present. It has the benefit of allowing trends to be reviewed and certain populations to be looked at specifically. In addition to the quality improvement approaches to the HIPE data system, it should be an ambition to institute a more robust method of surveillance in the future, aided by eHealth solutions.

### 4.1 Strengths and limitations

The accuracy of administrative data in quantifying these clinical events may not be optimal. Data relates to acute public hospitals only. Outpatient management of VTE is not captured in HIPE or other available data. HA-VTE occurring after discharge resulting in readmission is captured as a primary VTE rather than an HA-VTE. HA-VTE data may also exclude short in-patient admissions in which the patient is boarded, ie, due to congestion, no in-patient bed is available, resulting in some or all of the admissions not being recorded in HIPE data. HA-VTE data excluded maternity and pediatric admissions and hospitals, due to the different populations having very different occurrence rates of HA-VTE. Nonetheless, secondary PA-VTE codes were recorded in acute adult hospitals, and secondary VTE data related to neonates were also reported.

The HIPE data obtained was completely anonymized; therefore, in its current form, it is not suitable for longitudinal tracking to identify readmissions. The absence of patient-level linkage biases the incidence rate estimates, as the primary VTE crude incidence rate may be overestimated due to readmissions (which should be coded as secondary VTE). Most of Irish documentation is handwritten, and data extraction from this can be a challenge. Readmission to the same hospital with VTE as a primary diagnosis is not currently part of the HSE key performance indicators [16]. In other healthcare systems, linking to other data sources (eg, to primary care) enables identification of postdischarge VTE and/or VTE managed in ambulatory settings [44]. We foresee improvements in the sensitivity and specificity of administrative codes for VTE, with wider implementation of electronic health records and the likely introduction of a unique patient identifier, facilitating data linkage.

The all births category reported by the CSO refers to live births only (including <28 weeks) and therefore does not capture VTE events occurring early in pregnancy or after a spontaneous abortion or stillbirth [13].

The likelihood of a low PPV for HA-VTE rates must be considered when reviewing these results. HA-VTE rates are difficult to compare due to a lack of public reporting and differences in what is reported. Potential underestimation of cases due to the exclusion of readmissions and certain DVT events managed in an ambulatory manner are important limitations. By advocating for a stronger patient safety culture and improved safety monitoring system, it is anticipated that the reported HA-VTE rate may increase but not reflect an increase in the incidence of HA-VTE.

The 95% CIs were provided for cumulative years (2015-2022), given the manner in which the data was obtained. Data suppression affected access both to groups with <5 cases and where providing data on another group could be used to calculate the data for groups with <5 cases, to prevent potential identification of individuals. This limited some aspects of data analysis; hence, it was decided to exclude some codes. The codes included in this paper were selected after careful consultation with HIPE coders.

### 4.2 Conclusion

This report of national VTE crude incidence and epidemiologic data in Ireland has its main strength in its use of a large nationwide dataset for analysis. Hospital discharges were utilized to provide empirical evidence; this arose from the establishment of national VTE analysis systems and subsequently a National VTE Clinical Program. This study will provide crucial baseline data to guide future prevention and treatment initiatives.
