# Supplementary material for: National trends in venous thromboembolism incidence in Ireland (2015-2022): a retrospective analysis
Source: Res Pract Thromb Haemost. 2026 Jun 12;10(5):106798. doi: 10.1016/j.rpth.2026.106798 (PMC13471209; doi:10.1016/j.rpth.2026.106798)
Supplement: Supplementary file 1 [file mmc1.docx]

|  | PE | DVT | PA-VTE | Total VTE |
| --- | --- | --- | --- | --- |
| Female (Percentage) | 7010 (51.2) | 7616 (51) | 658 (100) | 15,284 (52%) |
| Mean age (Y; SD; 95% CI) | 63 (0.53; CI 62-63) | 59.6 (0.6; CI: 59-60) | 32.7(1; 31.8 – 33.5) | 61 (0.58; CI 60.6-61.5) |
| Average LOS in days (IQR) | 7.5 (7.4 – 7.8) | 3.8 (3.7 - 4.0) | 3.1 (2.4 – 3.8) | 5.6 (5.4 – 5.7) |
|  |  |  |  |  |
| ICU admissions  (Percentage of Primary VTE) | 1344 (9.8%) | * | ~ | 1598 (18%) |
| Average LOS in days (IQR) (for patients with an ICU admission) | 13.4 (4 – 14.3) | - | - | 13.8 (12.1 – 14.8) |
| Average ICU LOS in days (IQR) | 4.5 (4.3 – 4.7) | * | - | 4.5 (4.1 – 4.8) |
| Death (Percentage of primary) | 413 (3) | 84 (0.6) | 0 | 497 (1.7) |

**Supplementary Table 1. Demographics: Adult primary VTE cases recorded in Ireland during 2015 – 2022**

PE: pulmonary embolism; DVT: deep vein thrombosis; PA-VTE: pregnancy-associated VTE. LOS: length of stay; ICU: intensive therapy/care unit.

For reasons of confidentiality the Healthcare Pricing Office does not disclose cells in which the number is ≤5, such cells have been replaced in this table with a tilde symbol (~). In cases in which further suppression was necessary to prevent suppressed cells from being disclosed such cells have been replaced with (*). Where counts are suppressed, summary statistics are also suppressed with (-).
